# Leptin-adiponectin imbalance as a marker of metabolic syndrome among Chinese children and adolescents: The BCAMS study

**DOI:** 10.1371/journal.pone.0186222

**Published:** 2017-10-11

**Authors:** Ge Li, Linxin Xu, Yanglu Zhao, Lujiao Li, Junling Fu, Qian Zhang, Naishi Li, Xinhua Xiao, Changhong Li, Jie Mi, Shan Gao, Ming Li

**Affiliations:** 1 Department of Endocrinology, Key Laboratory of Endocrinology, National Health and Family Planning Commission, Peking Union Medical College Hospital, Chinese Academy of Medical Science, Beijing, China; 2 Department of Endocrinology, The Frist Affiliated Hospital, Shanxi Medical University, Taiyuan, China; 3 Epidemiology Department, Fielding School of Public Health, University of California, Los Angeles, California, United States of America; 4 Division of Endocrinology, The Children’s Hospital of Philadelphia, Perelman School of Medicine, University of Pennsylvania, Philadelphia, Pennsylvania, United States of America; 5 Department of Epidemiology, Capital Institute of Paediatrics, Beijing, China; 6 Department of Endocrinology, Beijing Chaoyang Hospital, Capital Medical University, Beijing, China; Taipei City Hospital, TAIWAN

## Abstract

**Purpose:**

Leptin and adiponectin have opposite effects on subclinical inflammation and insulin resistance, both involved in the development of metabolic syndrome (MS). We aimed to investigate whether leptin/adiponectin ratio (L/A), as a marker of these two adipokines imbalance, may improve diagnosis of MS in children and adolescents, and determined its cut-off value in the diagnosis of MS.

**Methods:**

A total of 3,428 subjects aged 6–18 years were selected from Beijing Child and Adolescent Metabolic Syndrome study. Adipokine leptin and adiponectin were measured using enzyme-linked immunosorbent assay. Odds ratio of MS per 1 z-score of adipokine was examined using logistic regression. Diagnosis accuracy was assessed using c-statistics (AUC) and net reclassification index.

**Results:**

The levels of leptin and L/A increased with number of positive MS components, while the levels of adiponectin declined in both boys and girls (all *P* <0.001). AUCs for diagnosis of MS in girls were 0.793, 0.773, and 0.689 using L/A, leptin and adiponectin, respectively; and AUCs in boys were 0.822, 0.798, and 0.697 for L/A, leptin and adiponectin, respectively. Notably, L/A outperformed individual leptin or adiponectin in discriminating a diagnosis of MS (all *P* < 0.02 in AUC comparisons). In addition, the optimal cut-offs of L/A by ROC curve differed by genders and pubertal stages, which were 1.63, 1.28, 1.95 and 1.53 ng/ug for total, pre-, mid- and postpubertal boys, respectively and 2.19, 0.87,1.48 and 2.27 ng/ug for total, pre-, mid- and postpubertal girls, respectively, yielding high sensitivity and moderate specificity for a screening test.

**Conclusions:**

In this pediatric population, leptin-adiponectin imbalance, as reflected by an increase in L/A level, was found to be a better diagnostic biomarker for MS than leptin or adiponectin alone. Future longitudinal studies are needed to further validate the gender-specific cutoff values.

## Introduction

Metabolic syndrome (MS) is characteristic as a cluster of cardiometabolic risk factors, including central obesity, glucose intolerance, hypertension and dyslipidemia. MS is associated with insulin resistance (IR), type 2 diabetes (T2D) as well as cardiovascular disease (CVD) [1]. As in adults, obesity is the most common cause of IR in children, thereby the increasing prevalence of childhood obesity calls more attention to pediatric MS worldwide [2]. The available definitions of pediatric MS vary between guidelines [2, 3]. In the International Diabetes Federation (IDF) definition, abdominal obesity is an obligatory component, whereas in the most recent harmonized definition, abdominal obesity is only one of the key components [3]. However, not all overweight or obese children develop MS, T2D, or CVD. Many potential indicators beyond the traditional adiposity measures that may better reflect the function of adipose tissue, such as adipokines, are considered to be included in the expansion of MS definition and would hopefully provide further improvement for MS diagnosis [3, 4].

Among the numerous adipokines deprived from adipose tissue, adiponectin and leptin are crucial signal link between adiposity and metabolic disorders [5]. Leptin is not only a multifunctional metabolic regulator altering food intake, energy expenditure and neuroendocrine function, but also has been reported to be an important mediator of obesity related pro-inflammatory state that contributes to metabolic disorders [6]. On the other hand, adiponectin can improve the metabolic status via anti-inflammatory, improving insulin-sensitizing and anti-arteriosclerosis effects. Typical obesity in humans is commonly associated with elevation in leptin levels and decrease in adiponectin levels, suggesting that there must have been existed the imbalance of leptin-adiponectin regulation and this imbalance may play a role in the development of obesity related complications. In previous population studies, either hyperleptinemia or hypoadiponectinemia has been showed to be the early makers of metabolic disorders in both children and adults [5, 7–10]. Given that the distinct roles of leptin and adiponectin in cardiometabolic disorders, we hypothesized that imbalance of leptin-adiponectin regulation, as reflected by the circulating levels of leptin to adiponectin ratio (L/A), could potentially be a better indicator for diagnosis of MS than leptin or adiponectin alone. Indeed, several studies have provided such evidence in adults [11–18], but little was known in children and adolescents.

Therefore, leveraging the large cohort data from the Beijing Child and Adolescent Metabolic Syndrome (BCAMS) study [19], we compared the associations of serum leptin, adiponectin and L/A levels with MS and its components. Furthermore, we assessed the diagnostic value of L/A for MS and determined its optimal cut-off point among children and adolescents.

## Materials and methods

### Subjects

**Fig 1:**
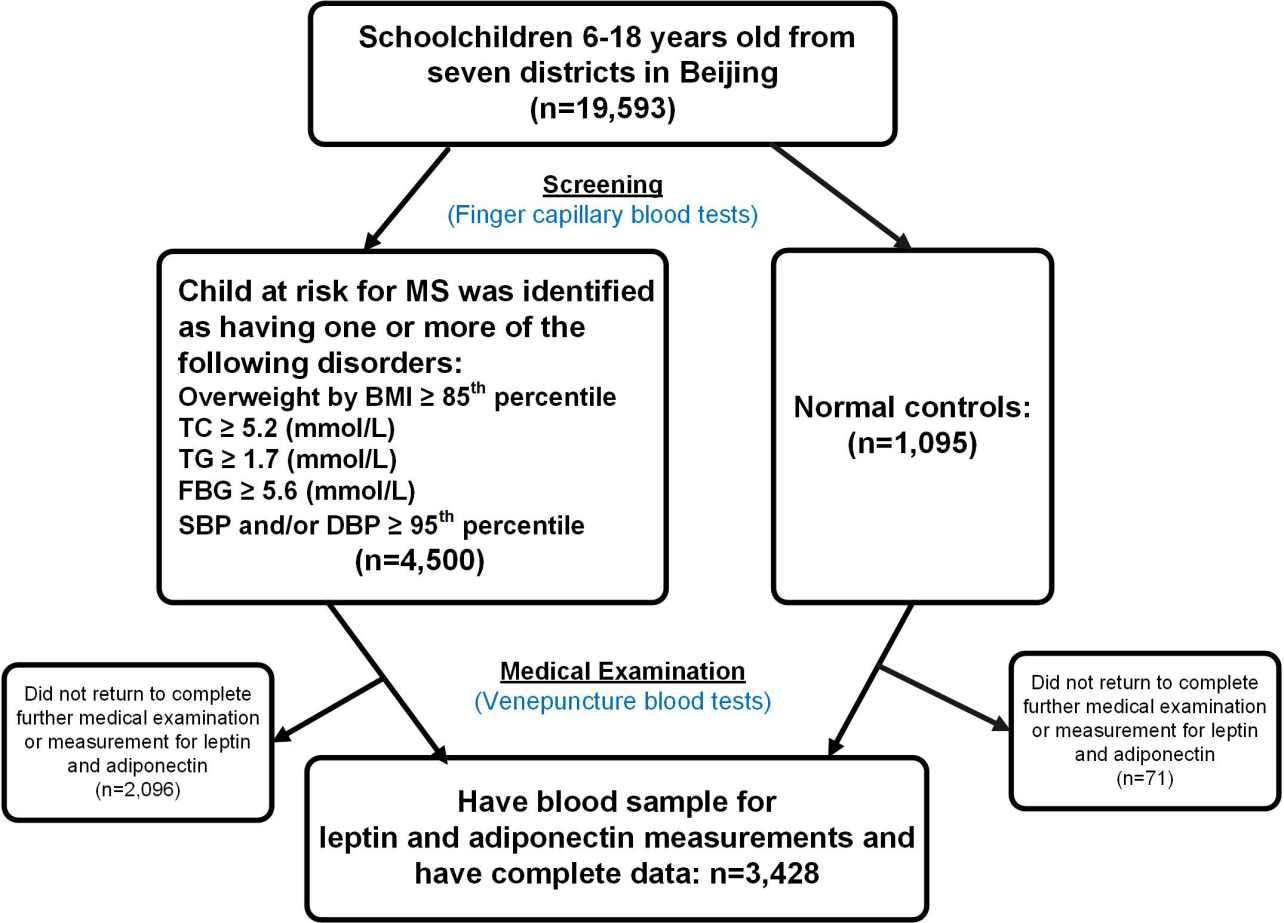
Flow chart.

The BCAMS study was designed as an ongoing follow-up study of obesity and related metabolic abnormalities (central obesity, hypertension, hyperglycemia and dyslipidemia) among a representative sample of school-aged children in Beijing (n = 19,593, 6 to18 years old, 50% male) [19]. Within this cohort, a total of 4,500 subjects were recognized as having one or more of the following disorders: being overweight, elevated blood pressure, increased total cholesterol (TC) ≥ 5.2 (mmol/L), triglyceride (TG) ≥ 1.7 (mmol/L) or fasting glucose (FBG) ≥ 5.6 (mmol/L) based on initial finger capillary blood tests. Further, all subjects at risk for MS, in parallel with a reference population of 1095 children, were invited to complete medical examinations for verification using venipuncture blood samples. In total, 3,428 subjects provided a blood sample for measurement of leptin and adiponectin and have complete data, thus were included in the current analysis (Fig 1). A detailed description of this cohort has been reported elsewhere [19]. The project was approved by the ethics committee of the Capital Institute of Pediatrics (approval number: 2012068). Written informed consent was obtained from all individual subjects and their parents included in the study. All experiments were performed in accordance with relevant guidelines and regulations.

### Clinical and anthropometric measurements

The weight and height of each child was measured according to the protocol that was used in our previous study [19]. Body mass index (BMI) was calculated by the formula weight (kg)/height (m^2^). Age- and sex-specific BMI percentiles, developed by the Working Group for Obesity in China, were used to define overweight (85^th^) and obesity (95^th^) [20]. Waist circumference (WC) was measured with a non-elastic tape measure. The WC measurement was taken at the end of expiration and in between the midpoint of the last rib and superior iliac crest. Systolic diastolic blood pressure (SBP) and diastolic blood pressure (DBP) were measured by a mercury sphygmomanometer with an appropriate sleeve for the patient’s age after a minimum of 20 min rest. The measurement was performed 3 times with an interval of at least 10 minutes, and the mean values of the latter 2 measurements were recorded as blood pressure. The National High Blood Pressure Education Program Working Group (2004) normal values for children were used as a reference to evaluate blood pressure measurements [21]. Pubertal development was scored by Tanner stage of breast development in girls and testicular volume in boys. A testicular volume equal to greater than 4 ml in boys and onset of breast development in girls were accepted as the criteria for onset of puberty [22]. This assessment was performed visually by two pediatricians of the same gender as the child. Subjects were also asked to complete questionnaires that included questions on physical activity and dietary information, and this was described in details elsewhere [23]. Physical activity was expressed as low (< 3 times/week) and moderate-to-vigorous (MVPA) (≥ 3 times/week).

### Biochemical analyses

Blood samples were obtained early in the morning after a 12-h fast. Samples were kept on ice and sent to the laboratory for analysis within 2–4 hours. Fractions were separated by centrifuge and frozen at -80°C. Levels of FBG, TG, high-density lipoprotein cholesterol (HDL-C), and low-density lipoprotein cholesterol (LDL-C) were assayed by the Hitachi 7060 C automatic biochemistry analysis system. Serum insulin, leptin and adiponectin levels were both measured using the sandwich biotin-avidin enzyme-linked immunosorbent assay (BA-ELISA), which was developed by the Key Endocrinology Laboratory of Ministry of Health, Peking Union Medical College Hospital [24–26]. Insulin assay had an inter-assay coefficient of variation (CV) of < 9.0% and no cross-reactivity to proinsulin (< 0.05%). The intra-assay and inter-assay CVs for leptin were < 7.4% and < 9.3%, respectively, and < 5.4% and < 8.5%, for adiponectin respectively. Insulin resistance index was calculated by homeostasis model assessment of insulin resistance (HOMA-IR) [HOMA-IR = fasting insulin (mU/L) × fasting glucose (mmol/L) / 22.5].

### Definition of MS

A modified Adult Treatment Panel III (ATP III) definition [1, 27, 28] that is more suitable for children, was employed in which MS was defined by the presence of three or more of the following five components: (1) central obesity defined as WC ≥ 90^th^ percentile for age and gender (established based on the BCAMS study); (2) elevated SBP and/or DBP ≥ 90^th^ percentile for age, sex and height (according to the BCAMS study); (3) hypertriglyceridemia defined as TG ≥1.24 mmol/L, equal to the 90th percentile of the reference population; (4) low serum HDL-C (Low-HDL) defined as ≤1.03 mmol/L, ≤ 5th percentile of the reference population and (5) impaired fasting glucose (IFG) defined as ≥ 5.6 mmol/L.

### Statistical analysis

All skewed distributions were natural logarithm transformed (ln) for analysis. Results were expressed as mean ± standard deviation (SD), if not otherwise mentioned. Since females have higher levels of leptin and adiponectin than males, we performed all analyses separately for males and females. ANOVA followed by Student-Newman-Keul’s (SNK) post hoc pairwise comparison was used for comparison of age and metabolic parameters in different groups. Chi-square test was used for the comparison of residence and physical activity and Kruskal-Wallis test was used for puberty. Relations between levels of leptin, adiponectin, L/A and the components of MS were analyzed by partial correlation analysis controlling age, pubertal stage, residence, physical activity and dietary score. Multivariate logistic regression models were used to estimate ORs for MS, and levels of leptin, adiponectin and L/A were converted to standard deviation units (Z-scores) before logistic regression analysis. The areas under the receiver operating characteristic (ROC) curve (AUCs), net reclassification improvement (NRI) and integrated discrimination index (IDI) of leptin, adiponectin levels and L/A were used to assess the diagnosis value of MS. The AUCs were compared using Medcalc statistical software version 16.2.0, while NRI and IDI were calculated using R version 3.4.1 (http://cran.r-project.org/). Youden index (sensitivity + specificity−1) was calculated to determine the optimal cut-off values for MS, and the original values with the maximum of Youden index was considered as the optimal cut-off points. All statistical analyses except NRI, IDI and AUCs comparison were performed using SPSS version 19.0 software for windows (SPSS Inc., Chicago, IL, USA). Two-sided P-value < 0.05 was considered as statistical significance.

## Results

The characteristics of the subjects with different numbers of MS components were shown in **Table 1**. In boys, subjects with more MS components were older, while there was no significant age difference in different groups of girls. With the increase of MS components, the subjects were more mature and more often rural residents in boys or in girls. Regarding the life-style factors, subjects with fewer MS components tend to have healthier diets and physical activity habits. Subjects with more MS components had higher levels of BMI, WC, FBG, TG, LDL-C, insulin, HOMA-IR, SBP and DBP, and lower levels of HDL-C. In addition, the levels of leptin and L/A increased dramatically with increasing number of MS components, while the levels of adiponectin declined remarkably in both boys and girls. Notably, compare to those without any MS components, L/A levels in MS subjects (with ≥ 3 MS components) increased by 16.1-fold in boys and 5.5-fold in girls, which is much greater than the individual changes of leptin or adiponectin levels.

**Table 1:** Different parameters among children according to the numbers of MS components.

	Number of MS components					
Variables		0	1	2	≥ 3	*P*
**Boys**						
n		590	512	384	257	
Age (y)		12 ± 3^a^	12 ± 3^a^	12 ± 3^a^	13 ± 3^b^	**< 0.001**
Tanner stage 1/2/3/4/5%		43.0/14.9/16.7/ 12.9/12.5 ^a^	37.8/19.1/15.9/ 11.3/15.9 ^a^	41.1/14.4/10.9/ 10.6/22.9 ^b^	28.9/13.8/17.7/ 12.5/27.2 ^c^	**< 0.001**
Residence (urban %)		79.6 ^a^	67.7 ^b^	63.3 ^b^	65.0 ^b^	**< 0.001**
Diet score		27.9 ± 4.4 ^a^	28.0 ± 4.3 ^a^	27.6 ± 4.4 ^a^	26.8 ± 5.0 ^b^	**0.003**
MVPA (%)		66.8 ^a^	62.2 ^a,b^	54.8 ^b^	52.5 ^b^	**< 0.001**
BMI (kg/m^2^)		19.2 ± 3.7^a^	22.6 ± 4.3^b^	25.9 ± 3.9^c^	29.0 ± 3.6^d^	**< 0.001**
WC (cm)		66.4 ± 9.9^a^	75.5 ± 12.2^b^	84.2 ± 10.7^c^	92.7 ± 9.4^d^	**< 0.001**
FBG (mmol/L)		5.0 ± 0.4^a^	5.2 ± 0.5^b^	5.2 ± 0.5^b^	5.4 ± 0.6^c^	**< 0.001**
TG (mmol/L)		0.72 ± 0.22^a^	0.96 ± 0.46^b^	1.31 ± 0.65^c^	1.64 ± 0.72^d^	**< 0.001**
HDL-C (mmol/L)		1.55 ± 0.32^a^	1.41 ± 0.32^b^	1.24 ± 0.24^c^	1.08 ± 0.21^d^	**< 0.001**
LDL-C (mmol/L)		2.41 ± 0.68^a^	2.50 ± 0.69^a^	2.68 ± 0.71^b^	2.74 ± 0.74^b^	**< 0.001**
Insulin (mU/L)*		5.18 (3.19–8.07)^a^	8.07 (5.53–12.14)^b^	11.13 (7.97–15.59)^c^	15.30 (11.12–23.37)^d^	**< 0.001**
HOMA-IR*		1.15 (0.69–1.83)^a^	1.89 (1.26–2.80)^b^	2.60 (1.83–3.68)^c^	3.62 (2.55–5.63)^d^	**< 0.001**
SBP (mmHg)		101.8 ± 12.1^a^	110.2 ± 12.7^b^	118.5 ± 12.8^c^	124.3 ± 12.0^d^	**< 0.001**
DBP (mmHg)		63.0 ± 8.9^a^	69.0 ± 9.3^b^	74.0 ± 9.1^c^	77.2 ± 8.7^d^	**< 0.001**
Leptin (ng/ml)*		1.47 (0.58–3.68)^a^	5.63 (1.78–12.21)^b^	10.11 (5.03–18.81)^c^	15.03 (8.03–25.82)^d^	**< 0.001**
Adiponectin(ug/ml)*		6.30 (4.25–9.28)^a^	5.47 (3.56–7.72)^b^	4.72 (3.27–6.85)^c^	3.64 (2.72–5.19)^d^	**< 0.001**
L/A(ng/ ug)*		0.23 (0.09–0.78)^a^	1.08 (0.31–2.74)^b^	2.12 (0.98–4.48)^c^	3.95 (2.08–7.63)^d^	**< 0.001**
**Girls**						
n		684	545	293	163	
Age (y)		13 ± 3	13 ± 3	13 ± 3	12 ± 3	0.333
Tanner stage 1/2/3/4/5%		21.7/13.7/13.9/ 37.9/12.7^a^	19.0/13.2/9.5/ 37.5/20.7^b^	17.3/11.8/12.8/ 32.2/26.0^b^	19.3/10.6/13.7/ 28.0/28.6^b^	**< 0.001**
Residence (urban %)		71.3 ^a^	54.4 ^b^	52.0 ^b^	53.7 ^b^	**< 0.001**
Diet score		28.3 ± 4.2 ^a^	27.3 ± 4.2 ^b^	27.3 ± 4.0 ^b^	27.5 ± 4.2 ^b^	**< 0.001**
MVPA (%)		57.3^a^	49.0 ^b^	44.6 ^b^	50.3 ^a,b^	**0.001**
BMI (kg/m^2^)		18.6 ± 3.1^a^	21.5 ± 4.0^b^	24.4 ± 4.2^c^	26.5 ± 4.2^d^	**< 0.001**
WC (cm)		62.7 ± 7.5^a^	69.6 ± 9.7^b^	77.3 ± 9.7^c^	81.9 ± 9.3^d^	**< 0.001**
FBG (mmol/L)		4.9 ± 0.4^a^	5.1 ± 0.5^b^	5.1 ± 0.7^b^	5.5 ± 1.4^c^	**< 0.001**
TG (mmol/L)		0.78 ± 0.21^a^	1.11 ± 0.55^b^	1.25 ± 0.57^c^	1.75 ± 0.90^d^	**< 0.001**
HDL-C (mmol/L)		1.55 ± 0.28^a^	1.40 ± 0.28^b^	1.26 ± 0.25^c^	1.10 ± 0.23^d^	**< 0.001**
LDL-C (mmol/L)		2.48 ± 0.72^a^	2.61 ± 0.77^a^	2.56 ± 0.71^a^	2.84 ± 0.72^b^	**< 0.001**
Insulin (mU/L)*		5.98 (3.71–8.75)^a^	8.8 (5.98–12.55)^b^	12.07 (8.51–16.97)^c^	14.63 (10.01–20.32)^d^	**< 0.001**
HOMA-IR*		1.31 (0.77–1.92)^a^	1.99 (1.32–2.77)^b^	2.75 (1.95–3.84)^c^	3.46 (2.32–5.15)^d^	**< 0.001**
SBP (mmHg)		99.1 ± 10.6^a^	105.8 ± 10.8^b^	112.4 ± 11.4^c^	117.0 ± 10.7^d^	**< 0.001**
DBP (mmHg)		63.1 ± 8.2^a^	67.6 ± 8.6^b^	71.1 ± 9.5^c^	75.6 ± 9.3^d^	**< 0.001**
Leptin (ng/ml)*		3.62 (1.67–7.59)^a^	8.44 (3.99–17.72)^b^	15.48 (7.45–26.63)^c^	16.35 (10.85–28.08)^d^	**< 0.001**
Adiponectin(ug/ml)*		6.63 (4.61–9.86)^a^	5.90 (4.04–7.96)^b^	4.78 (3.48–6.90)^c^	4.18 (2.96–6.18)^d^	**< 0.001**
L/A (ng/ug)*		0.58 (0.20–1.32)^a^	1.52 (0.59–3.47)^b^	3.27 (1.49–5.80)^c^	4.32 (2.23–8.39)^d^	**< 0.001**

Abbreviation: MVPA: Moderate-to-vigorous physical activity; BMI: body mass index; WC: waist circumference; FBG: fasting blood glucose; TG: triglycerides; HDL-C: high-density lipoprotein cholesterol; LDL-C: low-density lipoprotein cholesterol; HOMA-IR, homeostatic model assessment of insulin resistance; SBP: systolic blood pressure; DBP: diastolic blood pressure; L/A: leptin to adiponectin ratio; MS, metabolic syndrome.

0, 1, 2, ≥ 3 represented the number of MS components.

All values were reported as mean ± SD or median (interquartile range) or percentage. Significance was calculated by ANOVA followed by Student-Newman-Keul’s (SNK) post hoc pairwise comparison for age and metabolic parameters, or Chi-square test for residence and physical activity, or Kruskal-Wallis test for puberty. a, b, c and d meaned the difference between the two group after pairwise comparison. Variables with different letters were significantly different, with *P* < 0.05, that was, difference with the same letter were no statistical significant.

*Variables were ln-transformed before analysis.

Furthermore, partial association analysis was performed to investigate the relationships between circulating leptin, adiponectin, L/A levels and cardiometabolic parameters. In boys, BMI, WC, FBG, TG, LDL-C, ln-insulin, ln-HOMA-IR, SBP and DBP were all positively correlated with leptin levels after adjustment for age, puberty, residence and lifestyle-related factors, such as physical activity and dietary intake (all *P* < 0.001), while adiponectin levels were negatively correlated with the above parameters except FBG after adjusted for the same cofounders (all *P* < 0.01). Additionally, HDL-C levels were negatively correlated with leptin levels, and positively correlated with adiponectin levels in boys (all *P* < 0.001). For L/A, it was also positively correlated with BMI, WC, FBG, TG, LDL-C, ln-insulin, ln-HOMA-IR, SBP and DBP, and negative associated with HDL-C levels after adjusted for the above cofounders in boys (all *P* < 0.001). Similar trends were found in girls.

To further compare the associations of leptin, adiponectin and L/A with MS, logistic regression analysis was performed with adjustment for age, puberty, residence and lifestyle-related factors including physical activity and dietary intake. As show in **Table 2**, ln-leptin (boys: OR = 5.10 per SD, *P* < 0.001; girls: OR = 3.56, *P* < 0.001) and ln-adiponectin (boys: OR = 0.59 per SD, P < 0.001; girls: OR = 0.55 per SD, P < 0.001) were significant predictors of MS after adjusted for the above cofounders in both boys and girls. When further adjusted for BMI, the above ORs were moderately altered, but the significances of these associations except the one of ln-leptin in girls remained (all *P* < 0.01, except *P* = 0.080 for ln-leptin in girls). Furthermore, the adjusted odds ratio of MS per 1 SD increase of ln-L/A was 4.92 in boy (*P* < 0.001), and 4.54 in girls (*P* < 0.001) (**Table 2**). After further adjustment for BMI, ORs of MS were higher per 1 SD increase of ln-L/A (boys: OR = 2.03 (95CI%:1.51–2.72), *P* < 0.001; girls: OR = 1.81 (95% CI: 1.29–2.52), *P* = 0.001) than ORs of the ln-leptin (boys: OR = 1.81, *P* < 0.001; girls: OR = 1.32, *P* = 0.080) or the ln-adiponectin (boys: OR = 0.76, *P* = 0.003; girls: OR = 0.69, *P* < 0.001) in both boys and girls (**Table 2**).

**Table 2:** ORs and 95% CI for leptin, adiponectin and L/A with MS.

	Model 1			Model 2		
	OR	95%CI	*P*	OR	95%CI	*P*
**Boys**						
Ln-leptin z-score	5.10	3.96–6.57	**< 0.001**	1.81	1.32–2.46	**< 0.001**
Ln-adiponectin z-score	0.59	0.51–0.68	**< 0.001**	0.76	0.63–0.91	**0.003**
Ln-L/A z-score	4.92	3.87–6.24	**< 0.001**	2.03	1.51–2.72	**< 0.001**
**Girls**						
Ln-leptin z-score	3.56	2.80–4.52	**< 0.001**	1.32	0.97–1.81	0.080
Ln-adiponectin z-score	0.55	0.47–0.65	**< 0.001**	0.69	0.57–0.84	**< 0.001**
Ln-L/A z-score	4.54	3.49–5.92	**< 0.001**	1.81	1.29–2.52	**0.001**

Abbreviation: BMI: body mass index; Ln-leptin z-score: Ln-leptin z-score for per SD; Ln-adiponectin z-score: Ln- adiponectin z-score for per SD; Ln-L/A z-score: ln- (leptin to adiponectin ratio) z-score for per SD; MS, metabolic syndrome.

Model 1: adjusted for age, pubertal stages, residence, diet score and physical activity.

Model 2: Model 1+ additionally adjusted with BMI.

We then compared the value of L/A, leptin and adiponectin levels in the diagnosis of MS and its components (Table 3; Table 4). We found that L/A performed better than adiponectin alone in the diagnosis of central obesity, hypertriglyceridemia, elevated blood pressure, and better than leptin alone in the diagnosis of low HDL-C levels in both boys and girls, and the AUC of L/A also significantly improved in the diagnose of impaired fasting glucose in girls than using adiponectin alone. Moreover, L/A performed better than leptin and adiponectin levels individually in the diagnosis of MS in both boys and girls (all *P* < 0.05). AUCs in girls were 0.793, 0.773 and 0.689 for L/A, leptin, and adiponectin respectively, and AUCs in boys were 0.822, 0.798, and 0.697 for L/A, leptin, and adiponectin, respectively. Statistically significant increases were also observed for the net reclassification improvement and integrated discrimination index comparing models with L/A vs. models with either leptin or adiponectin levels (Table 4). Notably, the net reclassification improvement for model with was >13% with respect to the model with either leptin or adiponectin levels.

**Table 3:** The comparison of the value of leptin, adiponectin and L/A in the diagnose of MS and its components.

	Leptin (ng/ml)				Adiponectin (ug/ml)				L/A (ng/ug)			
	AUC (95% CI)	cut-off value	se (%)	sp (%)	AUC (95% CI)	cut-off value	se (%)	sp (%)	AUC (95% CI)	cut-off value	se (%)	sp (%)
Boys												
Central obesity	0.873 (0.856–0.888)	5.82	84.2	75.9	0.660 (0.638–0.683)	5.47	66.9	57.6	0.871 (0.854–0.887)#	1.08	85.3	73.9
Elevated blood pressure	0.694 (0.672–0.716)	3.67	78.1	53.8	0.587 (0.564–0.610)	6.14	69.8	45.8	0.694 (0.672–0.715)#	0.98	71.5	59.3
Hypertriglyceridemia	0.734 (0.712–0.754)	4.19	83.5	56.4	0.620 (0.597–0.642)	5.51	66.0	52.5	0.737 (0.716–0.758)#	1.10	76.3	61.5
Low HDL-C levels	0.629 (0.606–0.652)	2.63	83.4	39.4	0.702 (0.680–0.723)	4.47	69.1	65.2	0.682 (0.659–0.704)*	1.10	73.4	55.5
Impaired fasting glucose	0.511 (0.487–0.534)	1.33	79.5	25.3	0.508 (0.484–0.531)	7.19	72.7	30.9	0.509 (0.485–0.533)	0.16	84.4	21.6
MS	0.798 (0.773–0.822)	4.85	92.6	56.3	0.697 (0.664–0.729)	4.47	66.9	66.0	0.822 (0.799–0.845)*#	1.63	82.5	69.2
Girls												
Central obesity	0.843 (0.824–0.860)	9.11	78.2	76.2	0.655 (0.632–0.677)	7.07	80.7	43.7	0.835 (0.816–0.853)#	1.78	78.0	76.6
Elevated blood pressure	0.637 (0.613–0.660)	9.11	56.3	65.3	0.572 (0.549–0.596)	8.27	84.9	28.8	0.637 (0.614–0.660)#	1.34	62.8	58.7
Hypertriglyceridemia	0.604 (0.581–0.628)	8.97	56.9	60.8	0.606 (0.583–0.630)	4.79	48.6	68.9	0.668 (0.645–0.691)#	1.48	64.3	62.1
Low HDL-C levels	0.652 (0.629–0.675)	11.55	58.0	69.3	0.671 (0.649–0.694)	5.32	66.7	60.5	0.687 (0.664–0.709)*	1.95	64.4	64.9
Impaired fasting glucose	0.604 (0.581–0.628)	8.97	56.9	60.8	0.521 (0.497–0.545)	4.28	33.7	72.1	0.596 (0.572–0.619)#	1.59	58.7	59.8
MS	0.773 (0.740–0.806)	10.44	76.5	68.4	0.689 (0.650–0.728)	5.20	66.7	62.6	0.793 (0.761–0.825)*#	2.19	75.9	69.8

Abbreviation: L/A: leptin to adiponectin radio; MS: metabolic syndrome; AUC: area under the curve; se: sensitivity; sp: specificity.

* represents *P* values < 0.05 in the comparison of AUCs between leptin and L/A.

# represents *P* values < 0.05 in the comparison of AUCs between adiponectin and L/A.

**Table 4:** Comparison of adiponectin, leptin and L/A in the diagnosis of MS.

	AUC	*P*	NRI (%)	*P*	IDI (%)	*P*	Variables used for comparison
Boys							
Leptin (ng/ml)	0.798						
Adiponectin (ug/ml)	0.697	**< 0.001**	-28.65	**< 0.001**	-5.30	**< 0.001**	Leptin
L/A (ng/ug)	0.822	**0.001**	13.15	**0.010**	2.92	**0.001**	Leptin
		**< 0.001**	39.49	**< 0.001**	8.22	**< 0.001**	Adiponectin
Girls							
Leptin (ng/ml)	0.773						
Adiponectin (ug/ml)	0.689	**0.001**	-15.89	**0.022**	-3.10	**0.003**	Leptin
L/A (ng/ug)	0.793	**0.019**	42.72	**< 0.001**	1.92	**0.031**	Leptin
		**< 0.001**	19.20	**0.001**	1.18	0.100	Adiponectin

Abbreviation: L/A: leptin to adiponectin ratio; MS: metabolic syndrome; AUC: area under the curve; NRI: net reclassification improvement; IDI: integrated discrimination index.

The original values of leptin, adiponectin and L/A were used to calculate the AUCs, NRI and IDI.

Furthermore, as leptin and adiponectin levels might varied by different gender and age (or puberty puberty) (data not show), we determined the best threshold for L/A in the diagnosis of MS in each gender according to diverse pubertal stages (Table 5). Boys and girls were then separately divided into different groups according to Tanner stage for prepubertal (Tanner stage I) and midpubertal (Tanner stage II-III), postpubertal (Tanner stage ≥ IV) stages. As expected, optimum thresholds of L/A levels for the diagnosis of MS were 1.28 ng/ug (sensitivity = 88.1%, specificity = 64.2%), 1.95 ng/ug (sensitivity = 83.6%, specificity = 67.0%) and 1.53 ng/ug (sensitivity = 82.6%, specificity = 73.8%) for boys in pre-, mid- and postpubertal stages, respectively; and 0.87 ng/ug (sensitivity = 90.3%, specificity = 69.6%), 1.48 ng/ug (sensitivity = 82.1%, specificity = 66.5%) and 2.27 ng/ug (sensitivity = 83.1%, specificity = 63.1%) for girls from pre- to postpubertal stages, respectively.

**Table 5:** The cut-off values of L/A in predicting MS according to pre-, mid- and postpubertal stages.

	Prepuberty		Midpuberty		Postpuberty		All stages	
	boys	girls	boys	girls	boys	girls	boys	girls
Cut-off value (ng/ug)	1.28	0.87	1.95	1.48	1.53	2.27	1.63	2.19
Sensitivity (%)	88.1	90.3	83.6	82.1	82.6	83.1	82.5	75.9
Specificity (%)	64.2	69.6	67.0	66.5	73.8	63.1	69.2	69.8

Abbreviation: MS: metabolic syndrome; L/A: leptin to adiponectin ratio.

## Discussion

In this large cohort of children and adolescents, we confirmed that both increased leptin and decreased adiponectin levels were the independent risk factors of MS; moreover, we found that the associations of MS with the L/A levels were much stronger than those with leptin or adiponectin alone. In addition, we provide the first epidemiological evidence in Chinese pediatric population that L/A ratio was a potential better biomarker for diagnosis of MS than leptin or adiponectin alone, and the cut-off values of L/A varied by different gender and puberty. Our findings suggest that leptin-adiponectin imbalance, as reflected by an increase in L/A levels, may play an important role in the development of MS early in childhood.

Leptin and adiponectin, as two major adipokines derived from adipose tissue, have a broad spectrum of functions in the regulation of metabolism and are important link between obesity and MS [7, 29–31]. Individuals with obesity display markedly increased circulating leptin levels and decreased adiponectin levels, which suggests leptin resistance and adiponectin deficiency. In such cases, high leptin levels may also upregulate proinflammatory cytokines such as TNF-α and IL-6 that contribute to IR and MS [7, 18, 31]. In contrast, adiponectin has been identified as a key modulator with anti-inflammatory effects and adiponectin deficiency may lead to the occurrence of metabolic disorders [29, 30]. In 2005, it was recommended by IDF that both leptin and adiponectin, as the biomarkers of adipose tissue, should be included in the research to improve the diagnosis of MS [4, 32]. From then on, many epidemiological studies have been conducted in adults, providing evidence that supports the role of leptin and/or adiponectin as the novel biomarker for the diagnosis of MS [33–37]. In our pediatric study, we also confirmed that both increased leptin and decreased adiponectin levels were separately associated MS and its components, consistent with the similar existing studies in children [9, 10, 38, 39].

In addition, given that leptin and adiponectin have opposite effects on subclinical inflammation and IR, it was speculated that the combining use of those two adipokines, the L/A, may function as a better biomarker in the diagnosis of MS. Several cross-sectional studies in adults have compared the diagnostic value of L/A with that of leptin or adiponectin alone, and most studies supported the notion [11, 12, 14]. In contrast, a small study in adults with T2D reported that adiponectin performed better than L/A for the detection of MS [15]. Notably, a recent longitudinal study in Korean adults reported that L/A better predicted than adiponectin and leptin alone for the regression of MS in females and better than leptin in males [40]. However, evidence based on prospective studies remains scarce. Given the rising prevalence in pediatric obesity and MS, and the fact that children are otherwise relatively free of co-morbidities and usually treatment naïve, children constitute an interesting and valuable population to study the sequence of events contributing to MS-related pathology and find out the early biomarkers. However, pediatric study comparing the diagnostic value of L/A with that of leptin or adiponectin was still lacking. In a study of European children aged 2 to 9 years, L/A did not appear a better biomarker of MS as compared to leptin alone, although L/A was associated with MS stronger than adiponectin [38]. Inconsistent with these results, we found that levels of L/A had stronger association with MS than adiponectin or leptin separately, especially when controlling for BMI in our large cohort of children. These discrepancies may be due to the age, BMI and ethnic differences in the study populations. Furthermore, our analysis using ROC and NRI both confirmed that L/A had better ability for classifying MS children than adiponectin or leptin alone. These results suggest that the imbalance of leptin-adiponectin may be an important early mediator of MS development, thereby, correction of leptin-adiponectin imbalance maybe a novel target for early prevention and treatment of MS.

For clinical practice, we also evaluated the optimal cut-off points of leptin, adiponectin and L/A for diagnosis of MS based on ROC analysis. As reported in previous studies, all those thresholds displayed a significant gender specific difference. For instance, the best threshold of adiponectin for the diagnosis of MS is 4.5 ug/ml for boys and 5.2 ug/ml for girls in our study, which is comparable to the report from a study of Japanese, where male adult with a low adiponectin level (< 4 ug/ml) had a significant increased risk for CVD [41]. Moreover, as we observed leptin and adiponectin levels might varied by different puberty previously [42], we further analyzed the cut-off points in different pubertal stage. The cut-off value for L/A in prepubertal stage were slightly lower than those of pubertal stages in both boys and girls, though these cut-off values of L/A in our data for boys and girls in different pubertal stages were all slightly higher than the cut-off 1.00 ng/ug in males and 1.58 ng/ug in females in a study with relatively small samples in Mexican-American adults after translating their distinct units of adiponectin and leptin levels [12]. Our study provides new insight for the diagnostic ability of L/A for MS in different pubertal stages. However, attempting to define cut-off points for these adipokines in children can be challenging, since the reliable thresholds need longer-term outcome studies to validate.

The strengths and limitations of our study were listed as follows. To the best of our knowledge, this was the first study to establish the role of leptin-adiponectin imbalance in the diagnosis of MS in Chinese children and adolescents. In addition, our study is a large, well-characterized cohort of subjects with a range of metabolic traits and covariates measured. Accordingly, we were able to adjust many potential confounders documented to be correlated with both cardiometabolic risk and the adipokine levels, which in turn allows for our results to be particularly refined and robust. However, there are several limitations that should be noted. First, there is ongoing dispute about the definition of pediatric MS given the cut points for all ages throughout childhood in its criteria. Although we choose the modified ATP III criteria, the relatively suitable definition in pediatric setting, the use of different definitions in other subsequent pediatric studies may result in a little difficulty in comparing with our results. Second, the HOMA model was used to assess IR; however, the HOMA model is not the gold standard for assessing IR, although it provides good diagnosis value compared to the gold standard clamp techniques in validation studies of adolescents. Third, due to the cross-sectional design of this study, it is not possible to establish a causal relation between L/A and MS. Fourth, although as potential confounders, physical activity and dietary intake were assessed by using frequency questionnaires in our study, which might be relative weak compared with the metabolic equivalent of task (MET) and Healthy Eating Index (HEI) in the literatures [43, 44]. Finally, there are at least 3 forms of adiponectin in the circulation: low-molecular-weight, middle-molecular-weight, and the most active high-molecular weight (HMW) [5]. Since we only analyzed the total levels of adiponectin, further study are needed to confirm whether the leptin/ HMW adiponectin ratio will perform better than L/A in the diagnosis of pediatric MS. However, L/A was reported to be as effective as leptin/ HMW adiponectin ratio in adults [11]. Our on-going follow-up study of these children and adolescents may help to further clarify the usefulness of these biomarkers and reveal the best threshold.

Taken together, in this pediatric study, our results demonstrate that leptin-adiponectin imbalance, as reflected by the increased circulating levels of L/A, is a stronger indicator for the MS than adiponectin or leptin alone. Future research should investigate the potential pathways that can be possibly exploited to improve L/A levels, especially by elevating adiponectin levels in subjects at risk of cardiometabolic disorders.

## Supporting information

S1 File STROBE checklist. (DOCX)

## Abbreviations

L/A: Leptin to adiponectin ratio
MS: Metabolic syndrome
T2D: Type 2 diabetes
CVD: Cardiovascular disease
BMI: Body mass index
WC: Waist circumference
SBP: Systolic blood pressure
DBP: Diastolic blood pressure
FBG: Fasting blood glucose
TG: Triglyceride
HDL-C: High-density lipoprotein cholesterol
LDL-C: Low-density lipoprotein cholesterol
HOMA-IR: Homeostasis model assessment of insulin resistance
ROC: Receiver operating characteristic
AUC: Area under the curve
NRI: Net reclassification improvement
IDI: Integrated discrimination index
